# A Comparative Evaluation of Hydroxycamptothecin Drug Nanorods With and Without Methotrexate Prodrug Functionalization for Drug Delivery

**DOI:** 10.1186/s11671-016-1599-y

**Published:** 2016-08-31

**Authors:** Fuqiang Guo, Zhongxiong Fan, Jinbin Yang, Yang Li, Yange Wang, Hai Zhao, Liya Xie, Zhenqing Hou

**Affiliations:** 1 Department of Physics, Changji University, Changji, 831100 China; 2 College of Materials, Xiamen University, Xiamen, 361005 China; 3 People’s Hospital of Xintai City, Xintai, Shandong 271200 China; 4 The First Affiliated Hospital of Xiamen University, Xiamen, 361003 China

**Keywords:** Self-targeted multi-drug co-delivery system, 10-Hydroxycamptothecin, Methotrexate, Controlled and sustained release

## Abstract

We developed a novel self-targeted multi-drug co-delivery system based on rod-shaped 10-hydroxycamptothecin (CPT) nanoanticancer drug (CPT NRs) followed by a surface functionalization with self-targeting PEGylated lipid-conjugated methotrexate (MTX) pro-anticancer drug. The self-targeting effect and in vitro cell viability of the MTX-PEG-CPT NRs on HeLa cells were demonstrated by comparative cellular uptake and MTT assay of the PEG-CPT NRs. In vitro studies showed the feasibility of using this high drug-loading MTX-PEG-CPT NRs in self-targeted drug delivery, controlled-/sustained-release, and synergistic cancer therapy. More importantly, this work would stimulate interest in the use of PEGylated lipid-conjugated MTX by introducing an early-phase tumor-targeting role and then driving a late-phase anticancer role for the highly convergent design of nanomulti-drug, which may advantageously offer a new and simple strategy for simultaneously targeting and treating FA receptor-overexpressing cancer cells.

**Electronic supplementary material:**

The online version of this article (doi:10.1186/s11671-016-1599-y) contains supplementary material, which is available to authorized users.

## Background

Combination therapy has been considered to be a promising strategy for cancer treatment [1, 2]. Nanoparticles with multi-therapeutic actions targeted to different action sites of target cell could more effectively reduce the blood/renal clearance, increase the tumor accumulation, and, more importantly, induce the apoptosis/death and inhibit the growth of cancer cells [3]. In recent years, nanoparticles with chemo-chemo, chemo-gene, chemo-thermal, and chemo-photodynamic combination therapies have attracted considerable attention [4]. Especially, thousands of nanoparticles with chem-chem combination therapy have been widely used in treatment of many types of tumor. For the combination cancer chemotherapy, to achieve the synergistic anticancer effect, overcome the drug resistance, and reduce the side effects, multi-anticancer drugs have been co-loaded within the nanoscaled drug carriers of inert carrier materials and co-delivered to the tumor. However, when these drug carriers with comparatively low drug-loading ability (typically < 20 %) were clinically used for cancer treatment, the considerable decrease of effective accumulation of drug would occurred at the tumor site; in addition, large amounts of inert drug carriers could impose additional burden on the patients such as dosage-related systemic toxicity, metabolism/excretion/degradation concern, and serious inflammation [5, 6].

Anticancer drug [7], anticancer drug-anticancer drug physical complex [8], and anticancer drug-anticancer drug chemical conjugate [9, 10] as a material to construct the drug nanocrystals or nanoparticles (carrier-free nanoscaled drug system, self-delivery nanodrug) without (or with little) addition of any inert carrier materials have become attractive and have demonstrated the superior performance in the field of drug delivery for cancer treatment. However, insufficient bioenvironmental stability [11] or poor-targeting efficiency seriously limited its further clinical application. Our previous study demonstrated that methotrexate (MTX) anticancer drug itself could also be utilized as a folic acid (FA) receptor-overexpressing cancer cell-specific “targeting ligand,” playing a dual role [12, 13]. Inspired and motivated by the unique advantage of drug self-constructed nanoparticles and potential merits of therapeutic agent with a dual role, one would envisage the possibility of designing a self-targeting and high-drug loading nanomulti-drug to simply achieve the more effectively and specifically cell-targeting effect and cell-killing efficacy against cancer cell. The highly convergent design of self-targeting nanomulti-drug by “self-targeted multi-drug co-delivery and combination cancer therapy” may open a promising new door to nanomedicine.

## Results and discussion

**Fig. 1:**
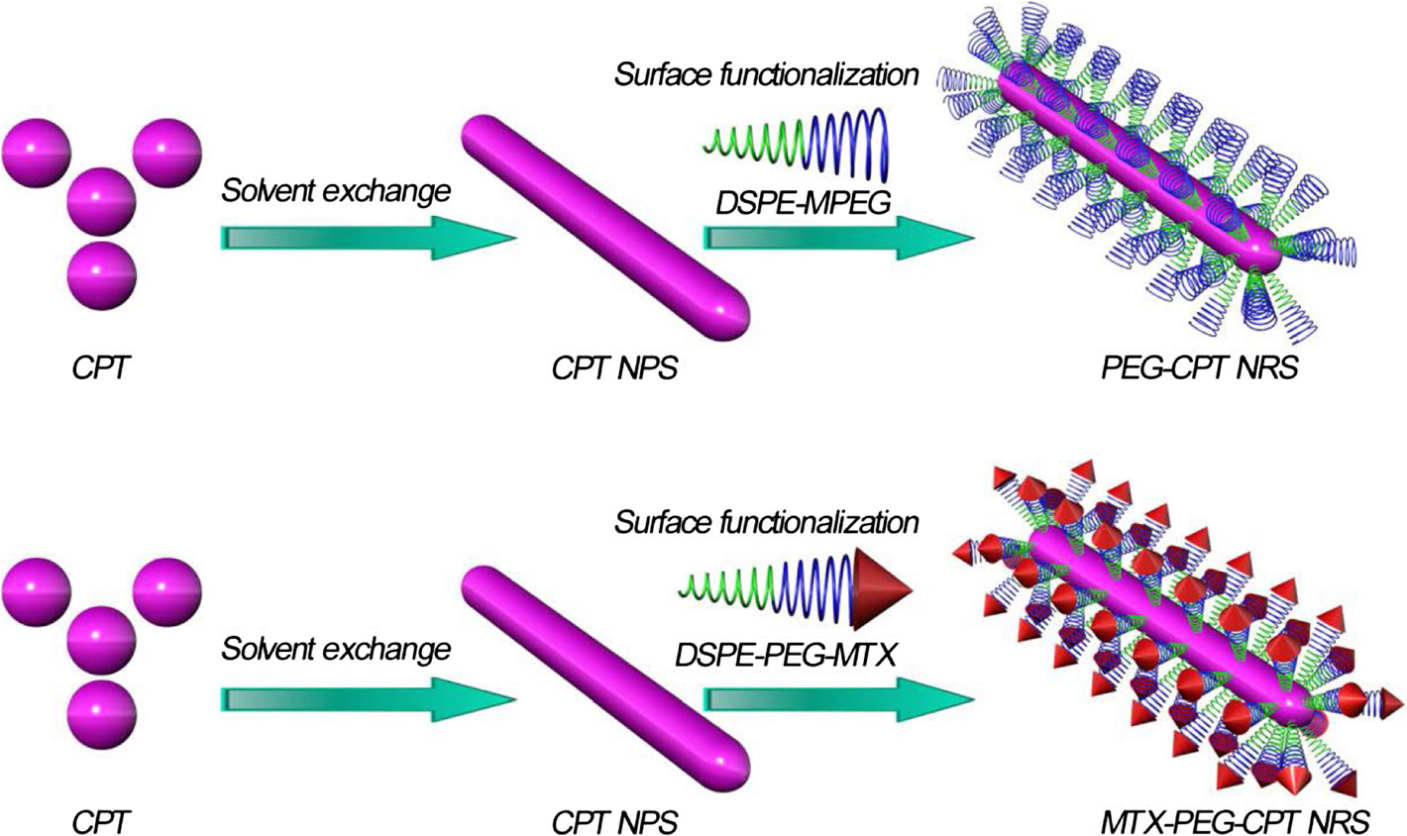
Schematic illustration of the construct of the CPT NRs by solvent exchange followed by the surface functionalization of DSPE-PEG-MTX or DSPE-MPEG (designed as MTX-PEG-CPT NRs or PEG-CPT NRs)

**Fig. 2:**
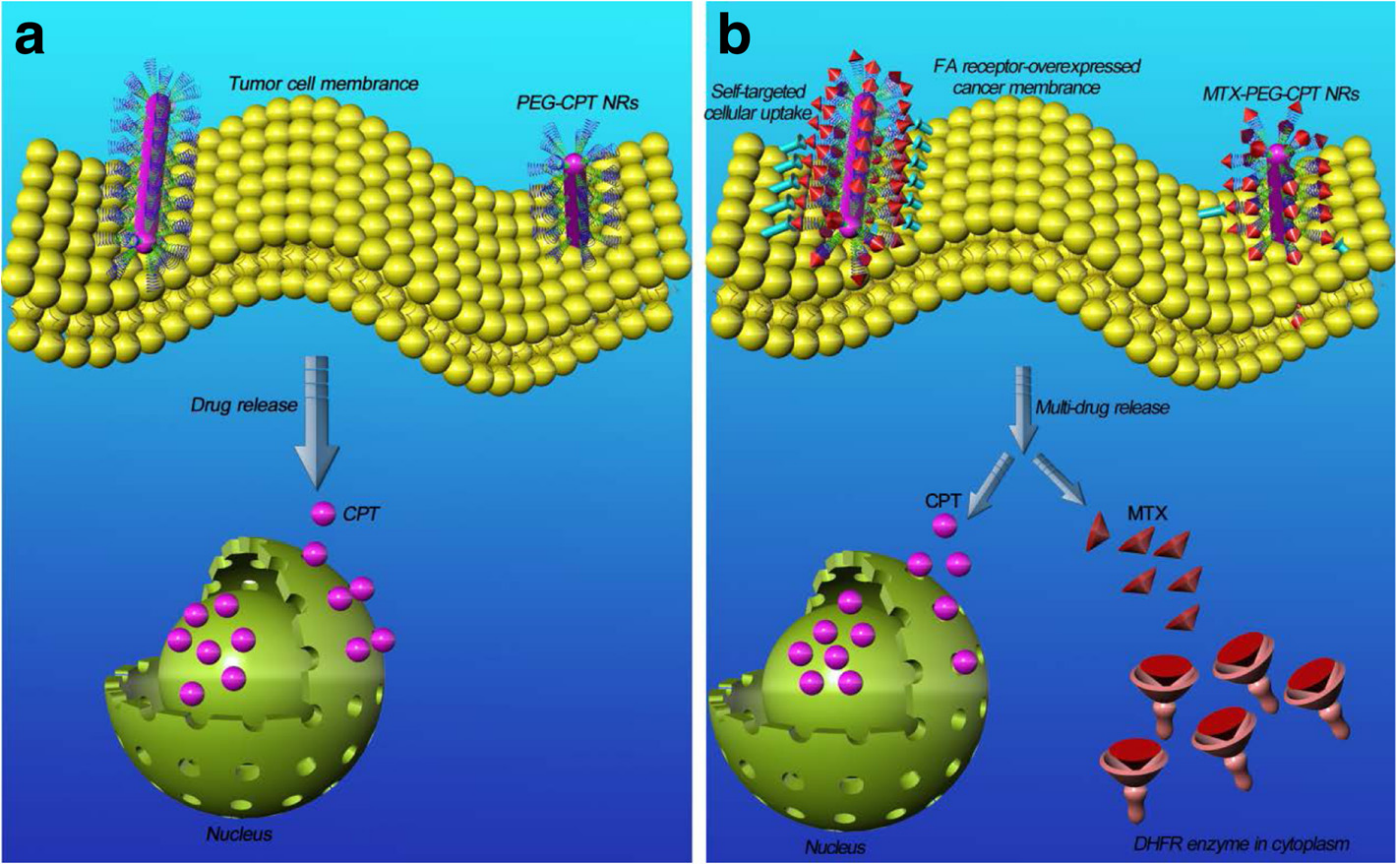
**a** Illustration of drug delivery of the PEG-CPT NRs. Once intravenously administrated, the PEG-CPT NRs were accumulated at the tumor site by the enhanced permeability and retention (EPR) effect-mediated passive tumor-targeting mechanism, were internalized by the tumor cells endocytosis, released CPT in a sustained manner, and intracellularly delivered CPT to the nucleus. **b** The MTX-PEG-CPT NRs with MTX self-targeting ligand enter tumor cells by FA receptor-mediated endocytosis and released both CPT and MTX anticancer drugs in tumor cells to be, respectively, delivered to nucleus and cytoplasm (CPT binds to DNA in the nucleus, and MTX binds to DHFR enzyme in the cytoplasm) for self-targeted multi-drug co-delivery and combination cancer therapy

Herein, we proposed a new strategy of preparing self-carried pure 10-hydroxycamptothecin (CPT) nanoparticles to form a hydrophobic drug (CPT) via solvent exchange followed by surface functionalization of self-targeting amphiphilic prodrug (Fig. 1) for self-targeted multi-drug co-delivery, cancer diagnosis, and combination cancer therapy (Fig. 2a, b). It is well known that each type of the drug has its unique inhibition mechanism or antitumor target [14]. For instance, MTX controls cancer by inhibiting dihydrofolate reductase (DHFR), which interferes with nucleic acid synthesis [15], while CPT controls cancer by inhibiting replication of DNA and transcription of RNA [16, 17]. Moreover, MTX could be not only utilized as an anticancer drug but also a cancer cell-specific targeting ligand because of its high structural similarity to FA. If both MTX and CPT drugs were combined in therapeutic treatment of FA receptor-overexpressing cancer cells, it is expected that their anticancer activity can be enhanced because of the self-introducing of targeting effect and achievement of synergistic anticancer efficacy of two types of drugs with different pharmaceutical functions, action modes, and anticancer mechanisms.

To obtain CPT-MTX composite nanomedicine, CPT was firstly dissolved in ethanol, and then, the mixture was added to water under ultrasonication for solvent exchange; finally, the resulted rod-shaped CPT drug nanoparticles (CPT NRs) were absorbed by DSPE-PEG-MTX (its structure was confirmed by ^1^H nuclear magnetic resonance (^1^H NMR, Additional file 1: Figure S1, ESI†), Fourier transform infrared spectroscopy (FTIR, Additional file 1: Figure S2, ESI†), and matrix-assisted laser desorption/ionization time of flight mass spectrometry (MALDI-TOF-MS, Additional file 1: Figure S3, ESI†) via hydrophobic interactions between the hydrophobic surface of NRs and the long-chain fatty acids of phospholipid of DSPE-PEG-MTX (designed as MTX-PEG-CPT NRs)). During the preparing process, ultrasonication was used to accelerate the diffusion of ethanol into water, resulting in the strong supersaturation and high nucleation rates to produce uniform nanosuspension. The addition of self-targeting PEGylated lipid prodrug to the hydrophilic region of the CPT NRs by non-covalent association resulted in the production of CPT NRs core-MTX shell-constructing MTX-PEG-CPT NRs with good water dispersibility, stability, and long-circulating ability.

**Fig. 3:**
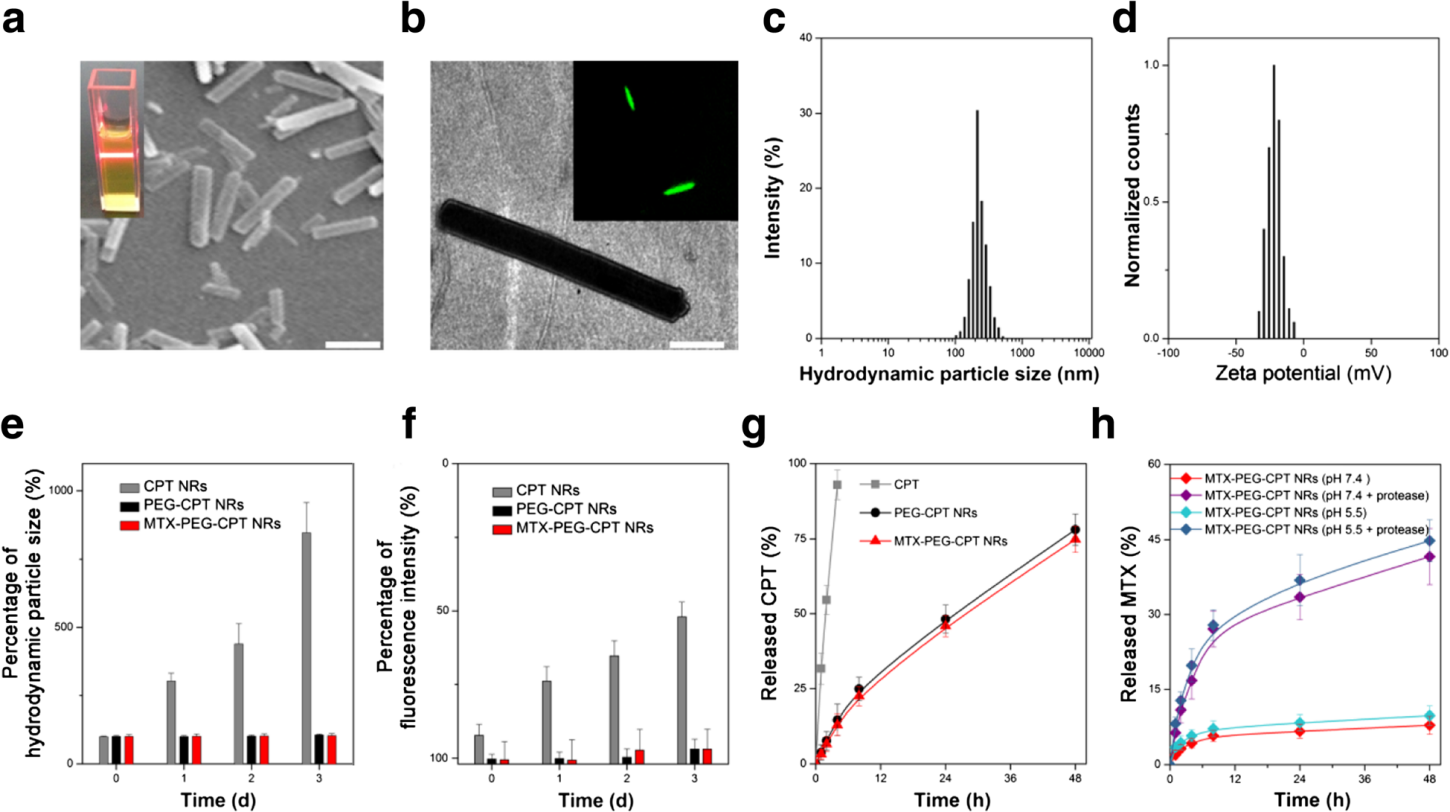
**a** SEM (*scale bars* 1 μm) (*inset* of **a**) tyndall effect. **b** TEM (*scale bars* 200 nm) (*inset* of **b**) LCSM image. **c** Hydrodynamic particle size distribution. **d** Zeta potential distribution of the MTX-PEG-CPT NRs. **e**, **f** In vitro physiological stability of the MTX-PEG-CPT NRs in PBS. **e** Hydrodynamic particle size change. **f** Fluorescence intensity change. **g**, **h** In vitro drug release of the MTX-PEG-CPT NRs. **g** CPT drug release. **h** MTX drug release. Data are presented as mean ± s. d. (*n* = 3)

The images observed by scanning electron microscopy (SEM) and transmission electron microscopy (TEM) showed the MTX-PEG-NRs were in dimensions of 1–2 μm/50–100 nm with rod shape (Fig. 3a, b). It was usually reported that the rod-shaped particles (higher aspect ratios) showed more obvious advantages compared with the sphere-shaped ones (lower aspect ratios) in vitro drug release, in vitro cellular uptake, and in vivo blood circulation/tumor accumulation [18–20]. The result determined by dynamic light scattering (DLS) and static light scattering (SLS) showed that the MTX-PEG-NRs had an average hydrodynamic particle size of nearly 250 nm with a narrow particle size distribution and a negative surface charge of about −30 mV (Fig. 3c, d). The CPT drug-loading content and MTX drug-loading content determined by high-performance liquid chromatography (HPLC) were, respectively, 74.8 ± 2.3 % and 2.6 ± 0.4 %. It was reported that the particle size of nanoparticles designed for in vivo drug delivery to tumor sites should be between 100 and 600 nm because vasculature of tumors varies from 100 to 600 nm [21]. Also, nanoparticles with positive surface charge and hydrophobic surface could promote protein adsorption and reticuloendothelial clearance [22]. Therefore, these physiochemical characteristics of the MTX-PEG-CPT NRs were favorable for reducing the inert material usage, achieving the good physiological stability, and acquiring the suitable passive tumor-targeting effect via the enhanced penetration and retention (EPR) effect, which simultaneously make drug delivery/cancer therapy combined with cancer imaging possible. In addition, the physiochemical characteristic including hydrodynamic particle size, surface charge, bioenvironmental stability (discussed below), and CPT drug-loading content was similar (Table 1); hence, the PEG-NRs could be used as a reference for in vitro and in vivo evaluations.

**Table 1:**
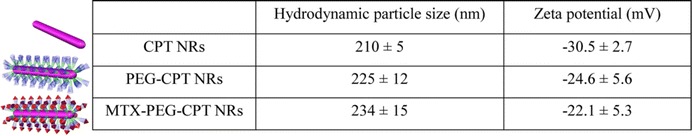
Hydrodynamic particle size, zeta potential, drug-loading content of the PEG-CPT NRs, and MTX-PEG-CPT NRs

Data are presented as mean ± s. d. (*n* = 3)

To evaluate the bioenvironmental stability of the MTX-PEG-CPT NRs, we determined the average hydrodynamic particle size and fluorescence intensity of the MTX-PEG-CPT NRs in phosphate-buffered saline (PBS) during 3 days of incubation period by DLS and fluorescence analysis (Fig. 3e, f). PEG-CPT NRs and MTX-PEG-CPT NRs show a no significant increase of average hydrodynamic particle size and decrease of fluorescence intensity. These results indicated the PEG-CPT NRs and MTX-PEG-CPT NRs possessed good bioenvironmental stability, whereas the CPT NRs observably form the large agglomerates and aggregation because of the charge elimination on the surface of CPT NRs by zwitterionic phosphonates of PBS. In addition, the MTX-PEG-CPT NRs also showed no significant change of hydrodynamic particle size and fluorescence intensity in Dulbecco’s modified Eagle medium with fetal bovine serum (10 %) (Additional file 1: Figure S4, ESI†). These results demonstrated the protecting effect of PEG on the MTX-PEG-CPT NRs against the zwitterion effect and protein adsorption by the combination of hydration effect and steric effect [23, 24].

The drug release profile of the MTX-PEG-CPT NRs is of great importance for multi-drug co-delivery applications. As shown in Fig. 3g, during the first phase of 8 h, the accumulative CPT release of the MTX-PEG-CPT NRs exhibited about 25 %. And both of these CPT NRs exhibited a similar CPT release behavior, which indicated the introduction of MTX-targeting ligand/anticancer drug did not alter the drug release of PEGylated CPT NRs with long-chain-length PEG. It was worthy noted that these two types of CPT NRs exhibited no early-phase serious burst release but a sustained and steady release characteristic. The result was explained by the fact that CPT with the crystalline state but not the amorphous one effectively avoids an unstable spatial arrangement, which is essential for their in vivo applications because of the reduced drug burst release in blood circulation, enhanced blood circulation effect, and improved tumor-targeted drug delivery.

We further studied the MTX release of the MTX-PEG-CPT NRs in PBS with different pH values in the presence or absence of proteases (pH 7.4 without proteases simulates blood circulation conditions, pH 5.0 with proteases simulates tumor cells lysosome). As depicted in Fig. 3h, at pH 7.4 in the absence of proteases, the accumulative MTX release of the MTX-PEG-CPT NRs at 24 h reached no more than 10 %. In sharp contrast, the addition of enzyme as well as the decrease of pH significantly promoted the MTX release from the MTX-PEG-CPT NRs. For instance, at pH 5.0 in the presence of proteases, the accumulative release of MTX greatly increased to about 45 % 24 h. This result indicated that the chemical linkage between MTX and MTX-PEG-CPT NRs was cleavaged by the combination of enzyme and pH to release the active MTX drug, “turn off” the targeting role of MTX while “turn on” the anticancer role of MTX. These also showed the potential for the in vivo applications such as the more effective multi-drug co-delivery to tumor microenvironment and tumor cells with reduced drug leakage/burst release in blood circulation.

**Fig. 4:**
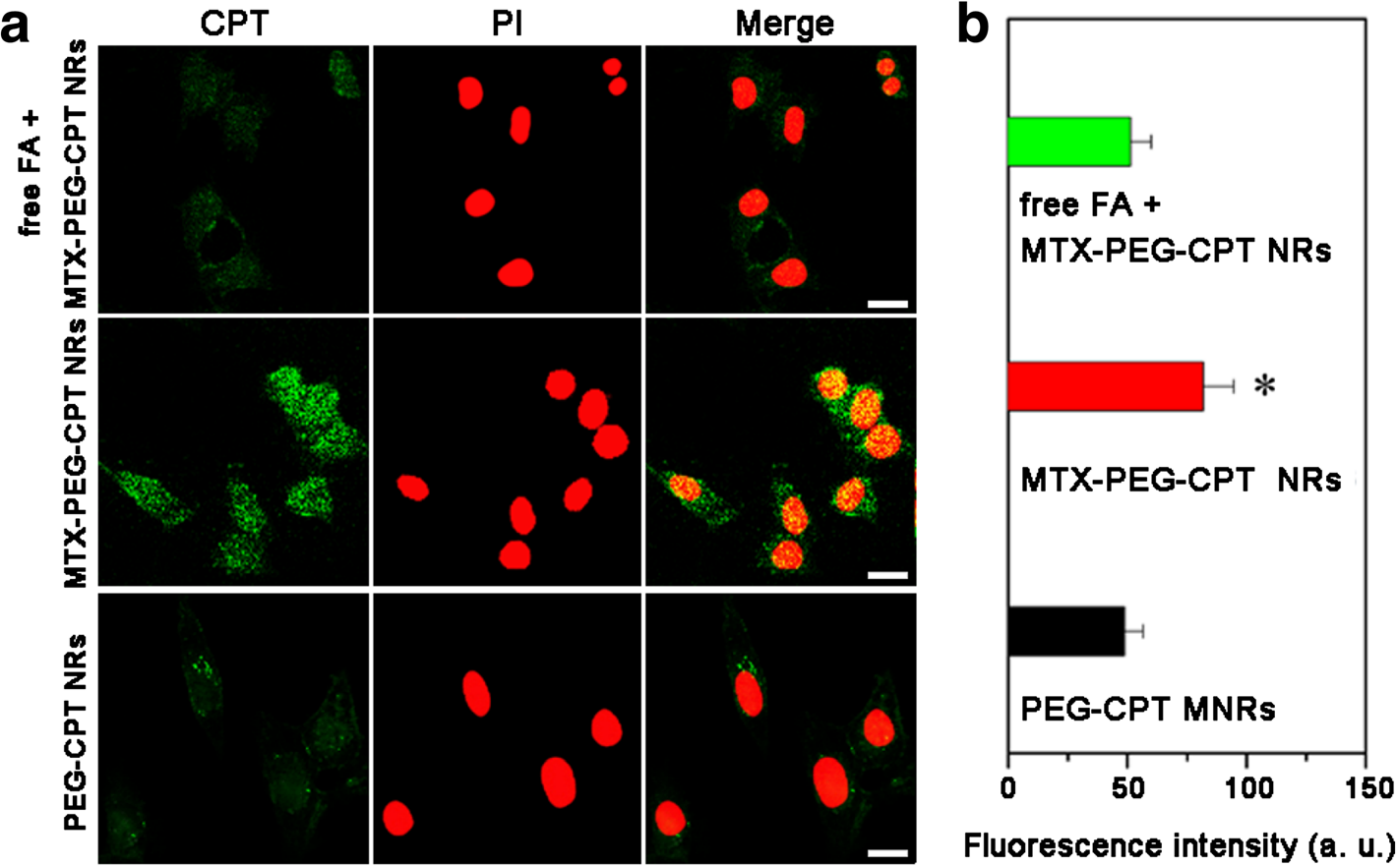
**a** CLSM images and **b** flow cytometry analysis of HeLa cells incubated with MTX-PEG-CPT NRs in the presence of excess of free FA, MTX-PEG-CPT NRs, and PEG-CPT NRs for 2 h. Cell nuclei are stained with PI. Data are presented as mean ± s. d. (*n* = 3). *P < 0.05 compared with the other groups

To verify the specificity of the MTX-PEG-CPT NRs for FA receptors, we performed a competition assay on HeLa cells overexpressed FA receptors. HeLa cells were pre-incubated with excess of free FA and then incubated with the MTX-PEG-CPT NRs. The cellular uptake of the MTX-PEG-CPT NRs by FA-treated HeLa cells was significantly reduced in comparison with untreated HeLa cells (Fig. 4). Consistent with our previous study [25, 26], the result suggested that MTX-functionalized nanomulti-drug could specifically bind to FA receptors overexpressed on the surface of HeLa cells.

We investigated the cellular uptake of the MTX-PEG-CPT NRs by HeLa cells using laser confocal scanning microscopy (CLSM). The qualitative result indicated that the functionalization of MTX improve the cellular internalization of the MTX-PEG-CPT NRs in comparison with the PEG-CPT NRs (Fig. 4). We further evaluated the cellular uptake of the MTX-PEG-CPT NRs by HeLa cells by flow cytometry. The quantitative results indicated the significantly higher cellular uptake efficacy of the MTX-PEG-CPT NRs compared with the PEG-CPT NRs (Fig. 4b). All results proved that self-targeting MTX moiety enhanced the target efficacy to potentially help the MTX-PEG-CPT NRs enter FA receptor-overexpressing HeLa cells by FA receptor-mediated endocytosis via multivalent receptor-ligand interactions [27].

**Fig. 5:**
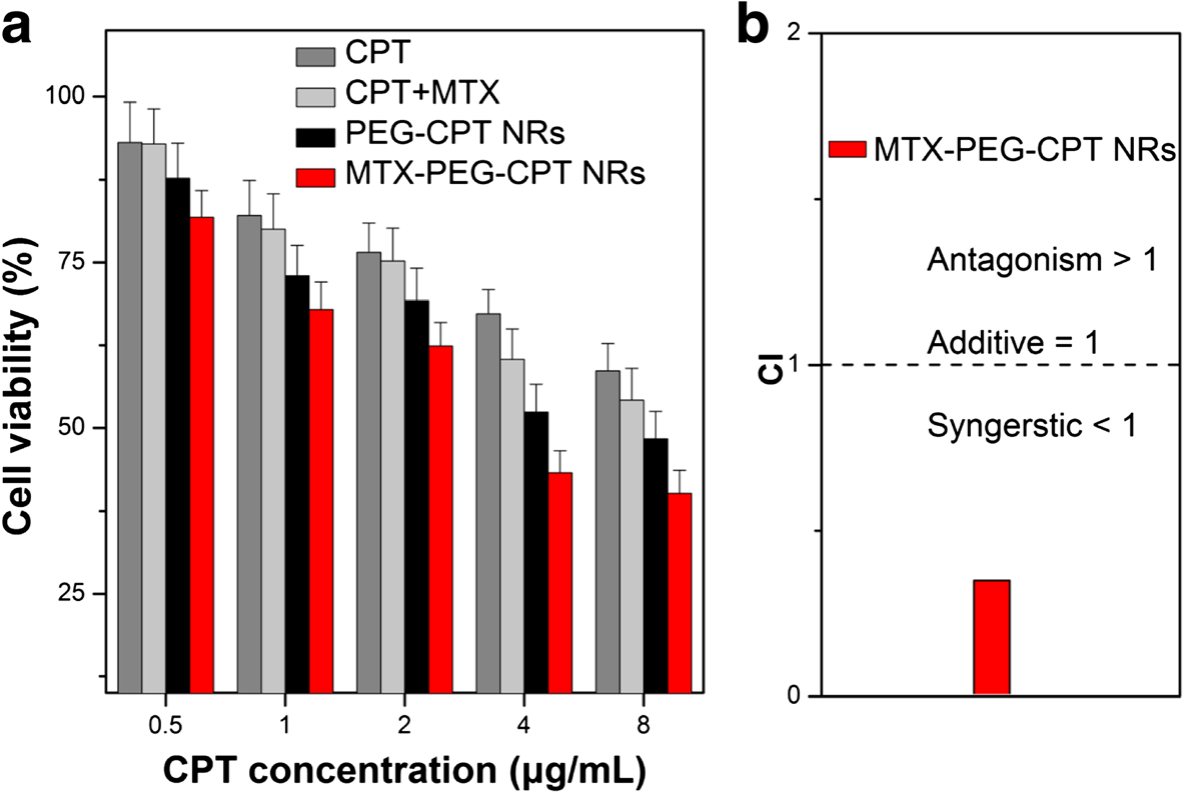
**a** Cell viability of HeLa cells incubated with the free CPT, free CPT plus free MTX, PEG-CPT NRs, and MTX-PEG-CPT NRs for 24 h of incubation. Data are presented as mean ± s. d. (*n* = 6). **b** Combination index for MTX-PEG-CPT NRs against HeLa cells

We next explored the in vitro cytotoxicity of the MTX-PEG-CPT NRs toward HeLa cells compared with the free CPT, free CPT plus free MTX, and PEG-CPT NRs by MTT assays. As shown in Fig. 5a, the MTX-PEG-CPT NRs showed much higher cytotoxicity than the free CPT, free CPT plus free MTX, and PEG-CPT NRs at the same CPT drug concentrations. The result demonstrated that the MTX-PEG-CPT NRs can obviously exert the inhibition effect of HeLa cell proliferation, as shown by a significant decrease in the half-maximal inhibitory concentration (IC_50_) values of the free CPT (IC_50_ = 9.9 μg/mL), free CPT plus free MTX (IC_50_ = 7.9 μg/mL), and PEG-CPT NRs (IC_50_ = 5.4 μg/mL) in comparison with the MTX-PEG-CPT NRs (IC_50_ = 3.2 μg/mL) after 24 h of incubation. The improved drug efficacy could be the more effective cellular uptake as well as sustained/controlled drug release of the self-targeting nanomulti-drug compared with either single-free drug molecule or both free drug molecule.

To further verify the drug synergistic effect of the MTX-PEG-CPT NRs, the combination index (CI), which could supply quantitative information regarding the extent of drug interactions, is calculated from the following equation [10, 28].

$$ \mathrm{C}\mathrm{I}=\mathrm{Nano}\hbox{-} \mathrm{Drug}1/\mathrm{Drug}1+\mathrm{Nano}\hbox{-} \mathrm{Drug}2/\mathrm{Drug}2 $$

where *Nano-Drug1* and *Nano-Drug2* are the concentration of the first drug and second drug of nanoparticles that in the combination to produce a certain effect (e.g., 50 % inhibition of cell viability). *Drug1* and *Drug2* are the concentration of single drugs to obtain the same effect. The CI value less than, equal to, or more than 1 is corresponding to the effect of synergism, additivity, and antagonism, respectively. As shown in Fig. 5a and Additional file 1: Figure S5, ESI†, the CI value of the MTX-PEG-CPT NRs was calculated as 0.35 (Fig. 5b), which indicated a highly synergistic effect of the MTX-PEG-CPT NRs’ both drugs acting on HeLa cell (CPT was released and delivered to the nucleus for inhibiting DNA activity, whereas MTX to the cytoplasm for inhibiting DHFR enzyme activity). All results demonstrated that the FA receptor-targeted, MTX-functionalized CPT NRs could specifically and efficiently enter the cancer cells to controlled and sustained release both CPT and MTX anticancer drug for achievement of greater anticancer efficacy and more effectively synergistic effect against HeLa cell.

## Conclusions

In summary, the self-targeting, controlled-/sustained-release, and multi-drug-loaded MTX-PEG-CPT NRs have been prepared for highly effective “self-targeted multi-drug co-delivery and combination cancer therapy.” The MTX-PEG-CPT NRs can be specifically uptaken by cancer cells, which result in an efficient intracellular both drug concentration and excellent cytotoxicity. More importantly, the MTX-PEG-CPT NRs can kill cancer cells through different functional roles, action sites, and anticancer mechanisms of both MTX and CPT, achieving a synergy in anticancer activity and showing a potential for clinical treatment of nanomedicine.

## Additional file

Additional file 1: Methods, general measurements, control experiments, additional table and figures. (DOC 2294 kb)
