# Supplementary material for: A Comparative Evaluation of Hydroxycamptothecin Drug Nanorods With and Without Methotrexate Prodrug Functionalization for Drug Delivery
Source: Nanoscale Res Lett. 2016 Aug 31;11(1):384. doi: 10.1186/s11671-016-1599-y (PMC5007229; doi:10.1186/s11671-016-1599-y)
Supplement: Additional file 1: — Methods, general measurements, control experiments, additional table and figures. (DOC 2294 kb) [file 11671_2016_1599_MOESM1_ESM.doc]

**Supplement Information**

**A Comparative Evaluation of Hydroxycamptothecin Drug Nanorods with and without Methotrexate Prodrug Functionalization for Drug Delivery**

Fuqiang Guo, ^1^ Zhongxiong Fan, ^1,2†^ Jinbin Yang, ^3^ Yange Wang, ^2^ Liya Xie, ^4^*Zhenqing Hou, ^1,2^*

^1^ Department of Physics, Changji University, Changji 831100, China.

^2^ College of Materials, Xiamen University, Xiamen 361005, China. E-mail: houzhenqing@xmu.edu.cn, Fax: +(86)592-2183058.

^3^ People’s Hospital of Xintai City, Xintai, Shandong 271200, China.

^4^ The First Affiliated Hospital of Xiamen University, Xiamen 361003, China.

^†^ Equal contributors.

**Email address:**

Fuqiang Guo: cjxyedu@163.com

Zhongxiong Fan: ZhongxiongFan@cju.edu.cn

Jinbing Yang: [862076764@qq.com](mailto:862076764@qq.com)

Yange Wang: wangyange@xmu.edu.cn.

Liya Xie: xly885@163.com

Zhenqing Hou: houzhenqing@xmu.edu.cn

**Materials and methods**

**Materials**

All chemical reagents were of analytical grade and used without further purification unless otherwise stated. Deionized (DI) water was used throughout. 10-Hydroxycamptothecin (CPT) was provided by Huangshi Pharmaceutical Co. Ltd (China). 1, 2-distearoyl-sn-glycero-3-phosphoethanolamine-N-[amino(polyethylene glycol)-3400] (DSPE-PEG-NH_2_) and 1, 2-distearoyl-sn-glycero-3-phosphoethanolamine-N-[methoxy(polyethylene glycol)-3400] (DSPE-MPEG) were purchased from Avanti Polar Lipids (Alabaster, AL, USA). Methotrexate (MTX) and folate (FA) were purchased from Bio Basic Inc. (Markham, Ontario, Canada). N, N’-dicyclohexylcarbodiimide (DCC) and N-hydroxysuccinimide (NHS) were purchased from Sigma-Aldrich (St. Louis, MO, USA). Dulbecco’s modified Eagle’s medium (DMEM) was from Sigma Chemical Corp. Penicillin-streptomycin and trypsin-ethylenediamine tetra-acetic acid (EDTA) were from Invitrogen. Fetal bovine serum (FBS) was purchased from Gibco Life Technologies (AG, Switzerland).

**Cell cultures**

Human cervical carcinoma cell line HeLa cells were was obtained from American Type Culture Collection (ATCC). HeLa cells were grown in FA-deficient DMEM (DMEM without FA) supplemented with 10% FBS and 1% penicillin/streptomycin. The cells were cultivated in an incubator (Thermo Scientific) at 37 ºC in the presence of 5 % CO_2_ for 24 h.

**Synthesis of DSPE-PEG-MTX**

DSPE-PEG-MTX was synthesized starting from DSPE-PEG-NH_2_ and MTX molecule. In a typical reaction, [methotrexate](javascript:void(0);) (8.2 mg, 18.0 µmoL), dicyclohexylcarbodiimide (3.7 mg, 18.0 µmol) and N-hydroxysuccinimide (2.1 mg, 18.0 µmol) were dissolved in [anhydrous](javascript:void(0);) dimethyl sulphoxide (DMSO) (400 µL). After 30 min, the solution containing the activated MTX was added to a DMSO solution (400 µL) containing TEA of DSPE-PEG-NH_2_ (14.5 mg, 3.6 µmol). The resulting mixture was allowed to react at room temperature for 72 h with continuous stirring under a nitrogen atmosphere. The resulting mixture was filtered to remove the white precipitation of dicylcohexyl urea (DCU) and precipitated in ice-cold ether. The crude precipitate was further washed by dichloromethane (DCM) five times to remove the excess, unreacted starting material, and then dried under vacuum. The product was dialyzed (molecular weight cut-off of 6000) against phosphate-buffered saline (PBS) solution for 24 h and against water for further 24 h. The solution was passed through a 0.22 µm filter to remove insoluble traces, lyophilized for 24 h, and stored at -20 °C.

**Preparation of the MTX-PEG-CPT NRs**

The MTX-PEG-CPT NRs was prepared by a solvent exchange method. 1 mg of CPT was dissolved in 1 mL of ethanol. 200 µL of CPT/ethanol as the organic phase was dropped into 10 mL of water as the aqueous phase using a microsyringe under sonication for 10 min. For PEGylation, 200 µL of DSPE-PEG-MTX in 4% ethanol aqueous solution was added to the resulted CPT NRs, and the mixture was further subjected to ultrasonic treatment for 5 min. After preparation, the MTX-PEG-CPT NRs suspension was centrifuged at 14000 rpm for 10 min, washed with water, and centrifuged again. The process was repeated for two more times to remove the residual organic solvent. The MTX-PEG-CPT NRs was dispersed in water via ultrasonication.

The PEG-CPT NRs was prepared using the identical procedure except that DSPE-PEG-MTX was replaced by DSPE-MPEG at the equivalent mass for addressing the specificity of the MTX functionalized NRs towards FA receptors.

**Characterization of the MTX-PEG-CPT NRs**

The hydrodynamic particle size and polydispersity index (PDI) was determined by dynamic light scattering (DLS) using a Malvern Zetasizer Nano-ZS (Malvern Instruments, Worcestershire, U.K.). The zeta potential was determined by electrophoretic light scattering (ELS) using a same equipment. The morphology was visualized using scanning electron microscopy (SEM, LEO 1530VP, Oberkochen, Germany). For the characterization of the synthesized DSPE-PEG-MTX or MTX-PEG-CPT NRs, the ^1^H NMR spectrum was determined on a Bruker AV400 MHz NMR spectrometer (Bruker, Billerica, MA, USA). The UV-vis absorption spectrum was recorded with a Perkin Elmer Lambda 750 UV-vis-near-infrared spectrophotometer (Perkin-Elmer, Norwalk CT). The fourier transform infrared spectrum was performed on a Bruker IFS-55 infrared spectrometer (Bruker, Zurich, Switzerland). The fluorescence spectrum was recorded with a FluoroMax-4 Spectrofluorometer (HORIBA Jobin Yvon Inc., NY, USA).

**Drug-loading content**

To measure the amount of CPT or MTX loaded within the MTX-PEG-CPT NRs, the lyophilized MTX-PEG-CPT NRs was dissolved in [anhydrous](javascript:void(0);) dimethyl sulphoxide (DMSO) by sonication to destroy the MTX-PEG-CPT NRs. After the filtration by 0.22 μm filter membrane, a part of the filtrate was analyzed for the determination of MTX or CPT drug loading content by a high-performance liquid chromatography (HPLC) method. The drug loading content of CPT or MTX was calculated using eqn (1).

$\text{Drug loading content (\%) }=\text{ }\frac{\text{Amount of drug in the MTX-PEG-CPT NRs}}{\text{Weight of the MTX-PEG-CPT NRs}}\text{ }\times\text{ }100\%$ (1)

**In vitro stability of the MTX-PEG-CPT NRs**

The in vitro stability of the MTX-PEG-CPT NRs was performed under different media by incubating the MTX-PEG-CPT NRs in water, phosphate buffer saline (PBS) solution and cell culture medium containing fetal bovine serum (FBS) for 3 days.

**In vitro drug release of the MTX-PEG-CPT NRs**

The drug release profile of CPT (or MTX) from the MTX-PEG-CPT NRs was evaluated in PBS solution (pH 7.4) at 37 °C. 1 mL of the MTX-PEG-CPT NRs was transferred into a dialysis membrane (molecular weight cut-off of 3000 Da) and then immersed into 49 mL of PBS (pH 7.4). The medium was kept at 37 °C with gentle shaking. At the selected time intervals, 1 mL of the release medium was withdrawn for fluorescence measurements and the release medium was replaced with an equal volume of fresh medium. The concentration of released MTX or CPT was determined by a HPLC method. The accumulative drug release of the MTX-PEG-CPT NRs was expressed as a percentage of the released drug. The drug release profile of the free drug and PEG-CPT NRs was used as controls for comparison. The cumulative release curve was calculated using eqn (2).

The cumulative release curve was calculated using eqn (2).

$\text{Cumulative release (\%) }=\text{ }\frac{\text{1 }\times\sum_{i=1}^{n-1} Ci+\text{50 }\times\text{ }Cn\text{ }}{\text{weight of drug in the MTX-PEG-CPT NRs}}\text{ }\times\text{ }100\%$ (2)

where *Ci* means the concentration of CPT or MTX drug in dialysate at *i* time.

**In vitro cellular uptake of the MTX-PEG-CPT NRs**

For quantitative study, confocal imaging of cells was performed using a Leica laser scanning confocal microscope (Leica Microsystems, Mannheim, Germany). Confocal imaging of CPT was performed using a laser with a peak wavelength of 405 nm as the excitation source. HeLa cells were cultured in 6-well plates with at a density of 1 × 10^5^ cells per well. The cells were incubated at 37 °C and 5% CO_2_ for 24 h. 100 μL of the MTX-PEG-CPT NRs were added to the cells for specific incubation periods. After incubation, the cells were washed with PBS, fixed with 4% paraformaldehyde, stained with propidium iodide (PI) and imaged. The cells incubated with the PEG-CPT NRs or MTX-PEG-CPT NRs in the presence of the free FA at the equivalent CPT concentration were used as controls for comparison.

**Flow cytometry analysis of the MTX-PEG-CPT NRs**

HeLa cells were seeded in 6-well plates with a density of 1 × 10^5^ cells/mL and incubated for 24 h, and then incubated with the MTX-PEG-CPT NRs for specific incubation periods. After harvesting with trypsin-EDTA, cells were washed with PBS, and the fluorescence was measured using a Beckman Coulter Cell Lab Quanta SC with excitation wavelengths of 405 nm. The cells incubated with the PEG-CPT NRs or MTX-PEG-CPT NRs in the presence of the free FA at the equivalent CPT concentration were used as controls for comparison.

***In vitro* cytotoxicity of the MTX-PEG-CPT NRs**

The *in vitro* cytotoxicity of the MTX-PEG-CPT NRs was measured by a 3-(4,5-dimethylthiazol-2-yl)-2,5-diphenyltetrazolium bromide (MTT) assay according to the manufacturer’s suggested procedures. HeLa cells were incubated with the MTX-PEG-CPT NRs at different CPT concentrations for 24 h at 37 °C and 5% CO_2_. The data were expressed as the percentage of surviving cells. The cells incubated with the free CPT, free CPT plus MTX, and PEG-CPT NRs at the equivalent CPT concentration were used as controls for comparison.

In addition, the combination index CI was calculated using eqn (3).

$\text{CI }=\text{ }\frac{\text{IC}_{50}\text{ of CPT in the MTX-PEG-CPT nanodrug}}{\text{IC}_{50}\text{ of CPT in the free CPT}}\text{ }+\frac{\text{IC}_{50}\text{ of MTX in the MTX-PEG-CPT nanodrug}}{\text{IC}_{50}\text{ of MTX in the free MTX}}\text{ }$(3)

***In vitro* stability of the MTX-PEG-CPT NRs**

The *in vitro* stability of the MTX-PEG-CPT NRs was performed under different media by incubating the MTX-PEG-CPT NRs in water, phosphate-buffered saline (PBS) solution and cell culture medium containing fetal bovine serum (FBS) for 3 d.

**Statistical analysis**

All experiments were repeated at least three times. All data were expressed as mean ± s.d.. Statistical tests were performed by the Student’s *t-*test and one-way ANOVA as well as the Non-parametric Kruskal-Wallis. Non-parametric Kruskal-Wallis tests were used due to the small number of results (n < 5) available for comparison. The statistical difference was considered to be significant when the *P* value was less than 0.05.


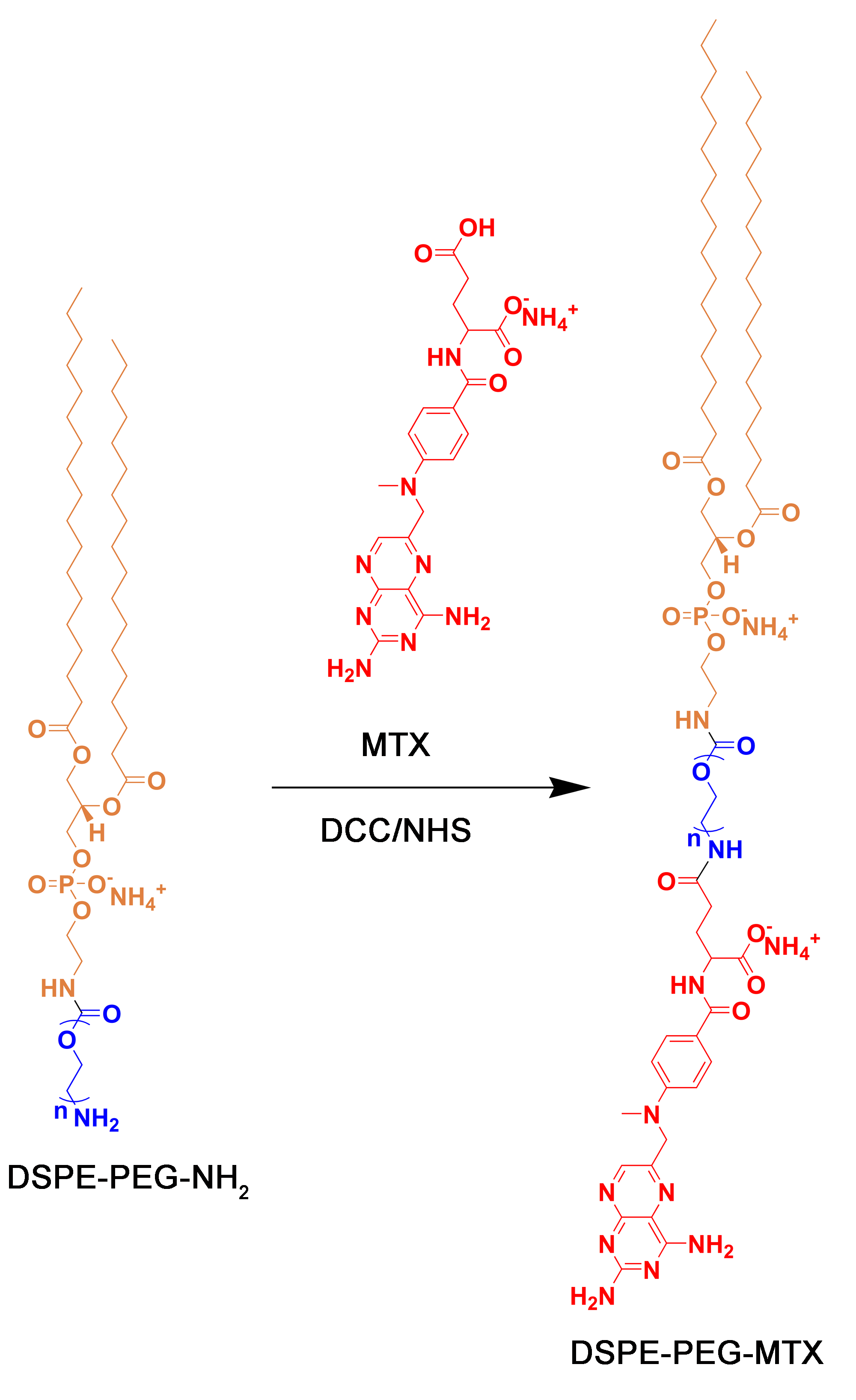


**Figure S1.** Synthetic route of self-targeting amphiphilic prodrug DSPE-PEG-MTX.


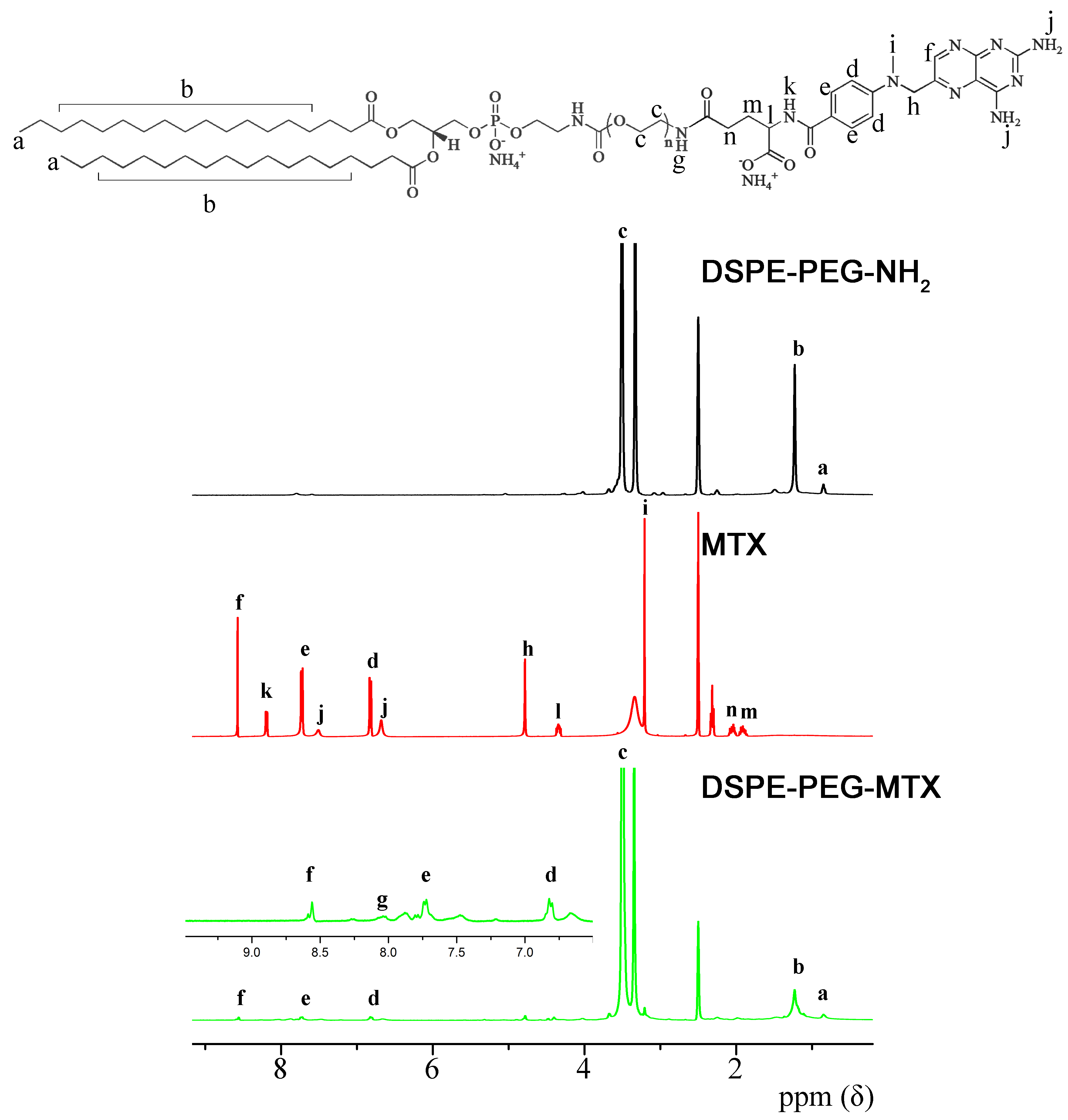


**Figure S2.** ^1^H NMR spectra of DSPE-PEG-NH_2_, MTX, and DSPE-PEG-MTX conjugate in DMSO-d6.

The characteristic peaks of the forming amide linkage between DSPE-PEG-NH_2_ and MTX were detected at 8.0 ppm. In addition, DSPE-PEG-MTX had a sharp proton peak of repeated units of PEG at 3.5 ppm, obvious proton peaks of methyl and methylene group of DSPE at 0.9 and 1.2 ppm, and characteristic proton peaks of pteridine ring and *p*-phenyl ring at 6.8, 7.8, and 8.6 ppm.


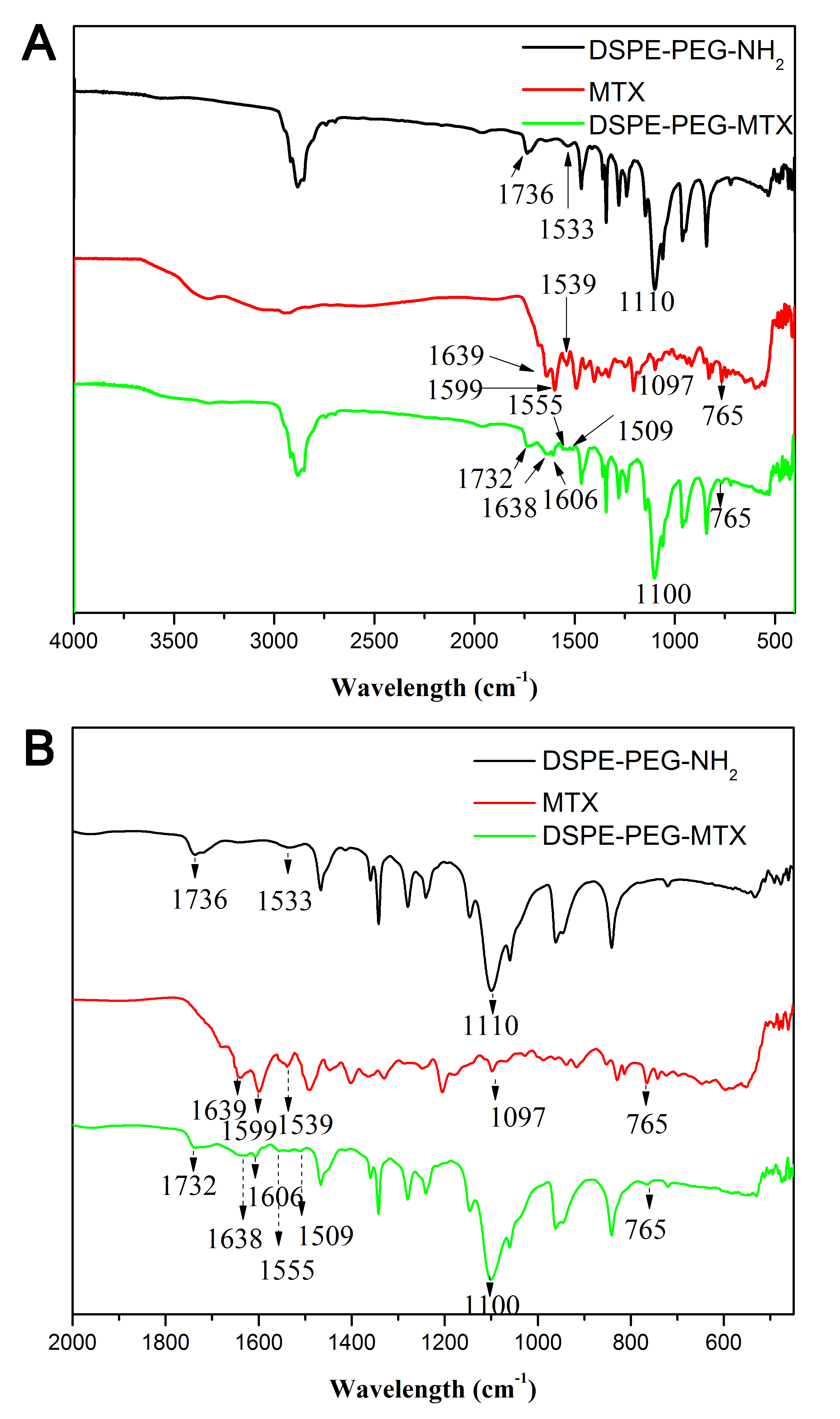


**Figure S3.** (A) ATR-FTIR spectra and (B) enlarged ATR-FTIR spectra of DSPE-PEG-NH_2_, MTX, and DSPE-PEG-MTX conjugate.

The ATR-FTIR spectrum of DSPE-PEG-MTX conjugate was shown in Figure S4. The new peak exhibited at 1732 cm^-1^ was attributed to the C=O bending vibrations, also the two characteristic peaks of DSPE-PEG-MTX at 1638 and 1555 cm^-1^ were respectively ascribed to ν(NC=O) (amide I) and δ(CN-H) (amide II) vibrations, indicating the formation of an amide bond between DSPE-PEG-NH_2_ and MTX. The strong peak exhibited at 1606 cm^-1^ was assigned to the typical N-H bending vibrations of primary amine of MTX. The peaks observed at 1509 cm^-1^ were due to aromatic skeletal vibration of benzene ring of MTX. The result demonstrated successful conjugation of MTX and DSPE-PEG-NH_2_.


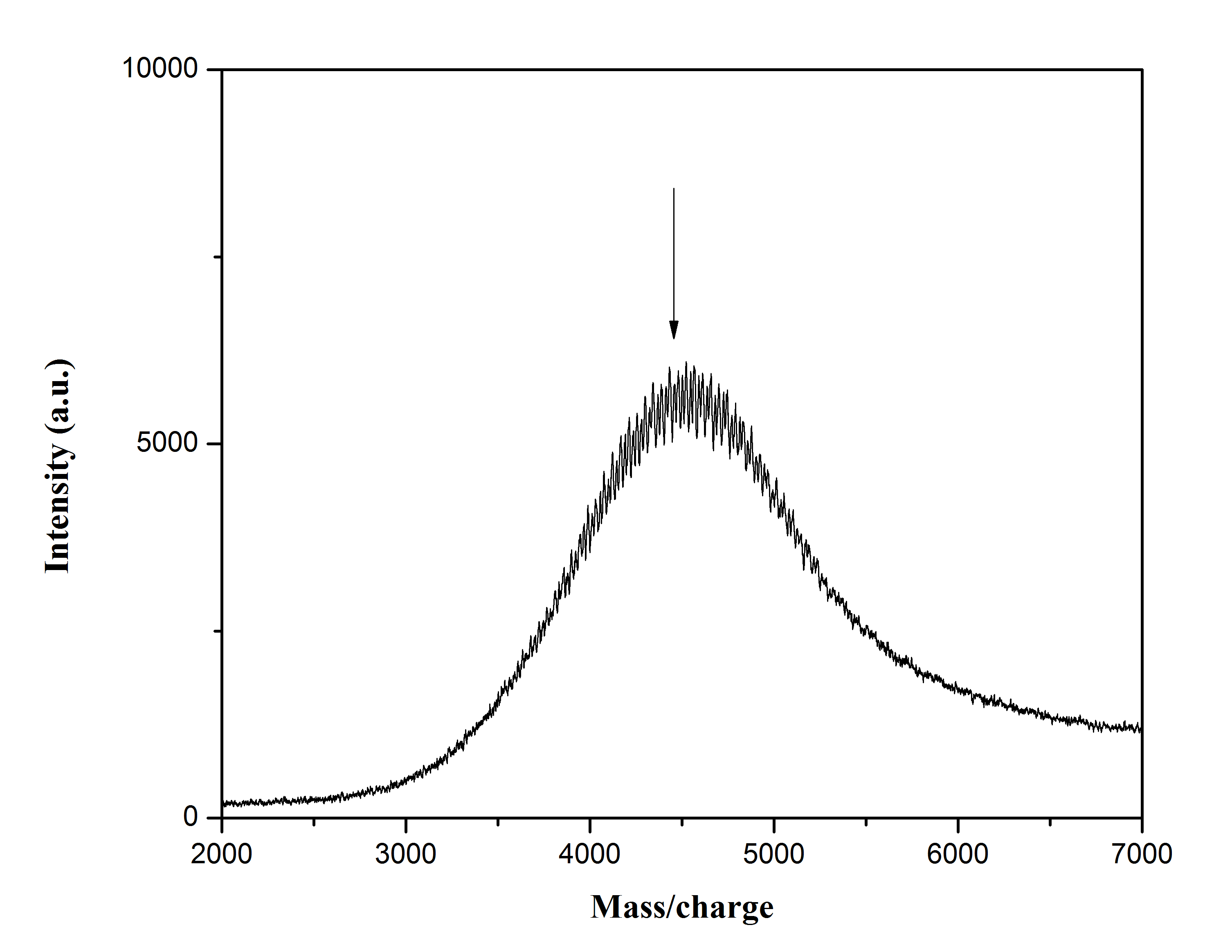


**Figure S4.** Matrix-assisted laser desorption/ionization time-of-flight mass spectrometer (MALDI-TOF-MS) analysis of DSPE-PEG-MTX. The arrow indicates the peak corresponding to the targeting compound. The experimental molecular weight of DSPE-PEG-MTX was determined to be approximately 4461 Da.


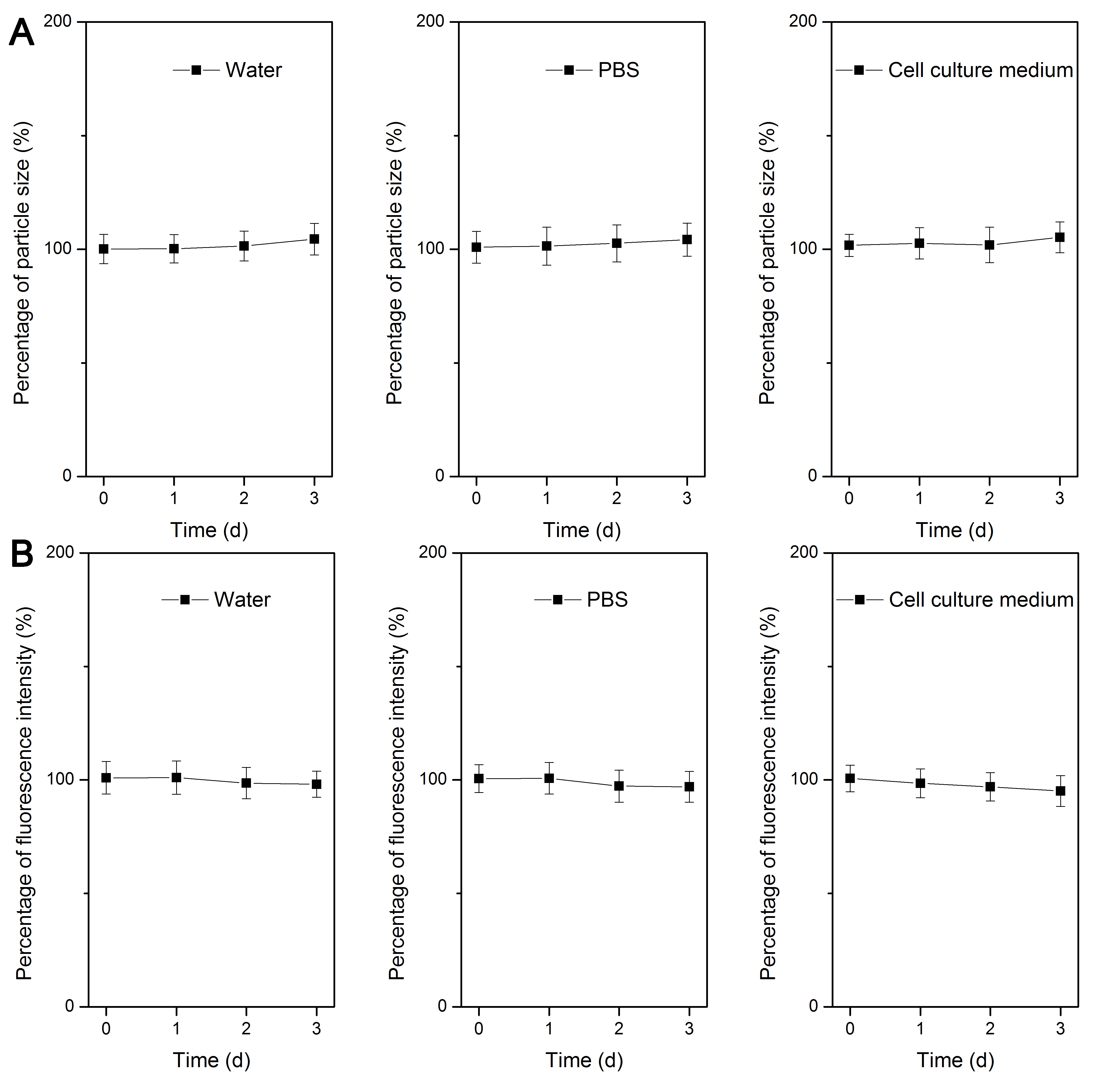


**Figure S5.** (A) Particle size change and (B) fluorescence intensity change of the MTX-PEG-CPT NRs dispersed in different physiological media determined by DLS and fluorescence analysis.


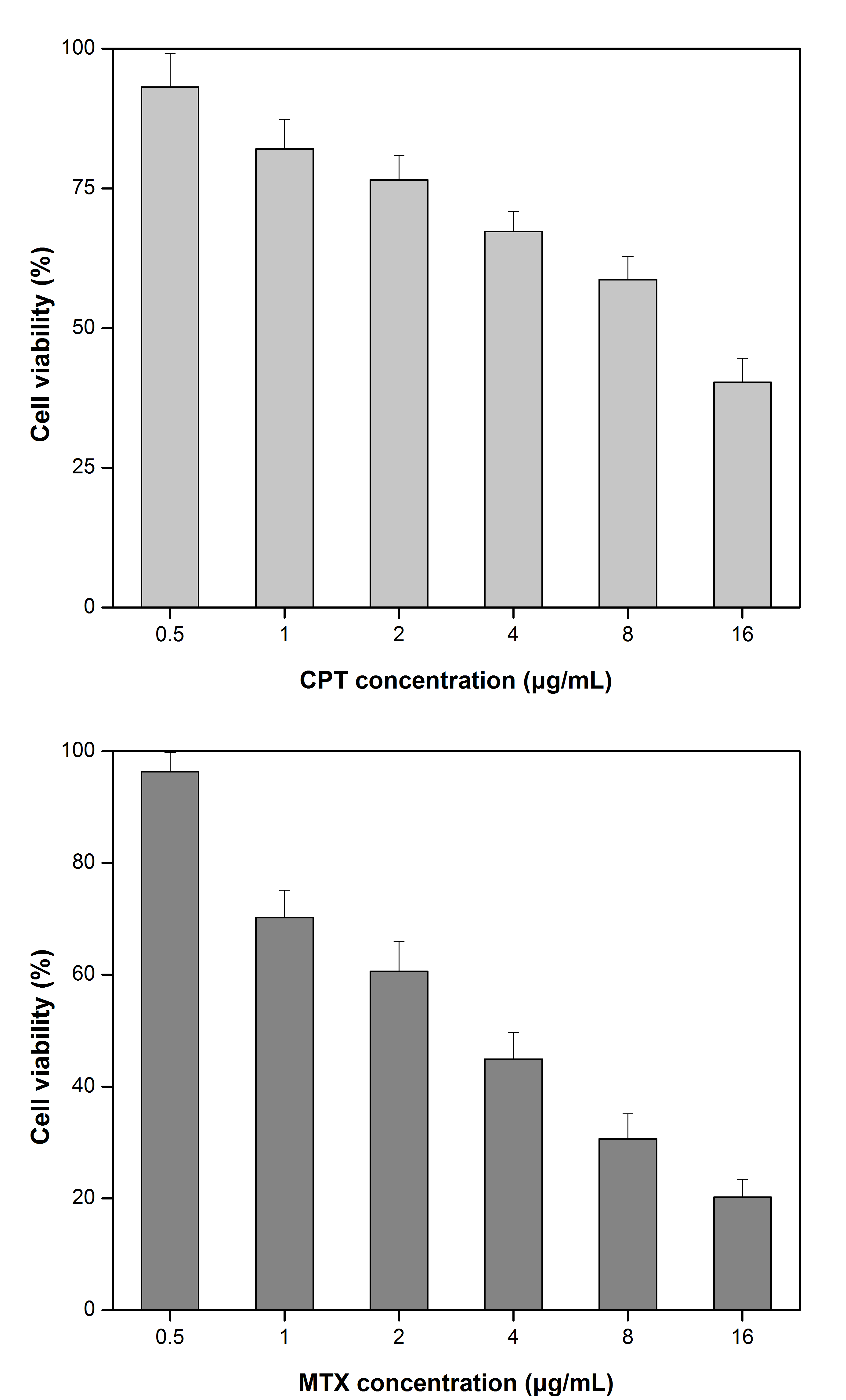


**Figure S6.** *In vitro* cell viability of HeLa cells treated with (A) free CPT or (B) free MTX after incubation of 24 h. Data are presented as mean ± s.d. (n = 6).


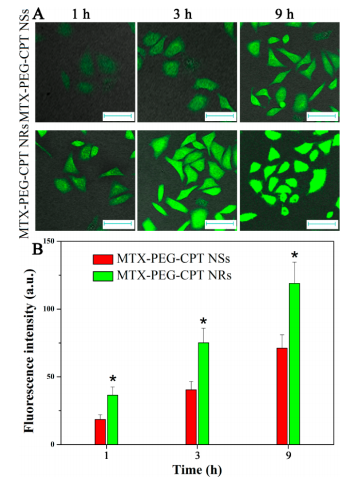


**Figure S7.** *In vitro* cellular uptake of the MTX-PEG-CPT NRs. (A) Confocal laser scanning microscopy images of HeLa cells incubatedwith the MTX-PEG-CPT NRs or MTX-PEG-CPT NSs for differentincubation times at 37 °C. All CPT concentration was equivalent. Allscale bars represent 25 μm. All images were taken under identicalinstrumental conditions by harmonizing the parameters such as laserpower, sensitivity, offset, and gain constant. (B) Fluorescencemeasurements of HeLa cells incubated with the MTX-PEG-CPTNRs or MTX-PEG-CPT NSs for different incubation times. Data are presented as mean ± s.d. (n = 6). *P < 0.05. [^1^](#_ENREF_1)

We investigated the cellular uptake efficiency of the MTXPEG-CPT NRs, HeLa cells were incubated with the MTX-PEG-CPT NRs and MTX-PEG-CPT NSs at the equivalent CPT concentration. The result of confocal scanning images (Figure S7A), fluorescence measurements (Figure S7B) presented the obviously higher cellular uptake efficiency of the MTX-PEG-CPT NRs over the MTX-PEG-CPT NSs. It was reported that shape could influence the targeting ability of the particles. [^2^](#_ENREF_2) Not only is the overall surface area available for targeting ligands important but also the local curvature also affects the degree to which particles fit the contours of target cell membranes. [^3^](#_ENREF_4) The improved cellular uptake of the MTX-PEG-CPT NRs was possibly explained by the shape-dependent effect. The much more efficient endocytosis induced by the more effectively multivalent specific ligand-receptor interaction opportunity between the elongated as well as targeted nanoparticles surface’s multivalent MTX ligands and the cell membrane surface’s multivalent FA receptors [^4^](#_ENREF_5) was responsible for the significant increase of the binding avidity and specificity of the MTX-PEG-CPT NRs toward HeLa cells.

**References**

1. Li, Y.; Lin, J.; Huang, Y.; Li, Y.; Yang, X.; Wu, H.; Wu, S.; Xie, L.; Dai, L.; Hou, Z., Self-Targeted, Shape-Assisted, and Controlled-Release Self-Delivery Nanodrug for Synergistic Targeting/Anticancer Effect of Cytoplasm and Nucleus of Cancer Cells. *ACS applied materials & interfaces* **2015,** *7* (46), 25553-9.

2. (a) Park, J. H.; von Maltzahn, G.; Zhang, L.; Schwartz, M. P.; Ruoslahti, E.; Bhatia, S. N.; Sailor, M. J., Magnetic Iron Oxide Nanoworms for Tumor Targeting and Imaging. *Advanced materials* **2008,** *20* (9), 1630-1635; (b) Kolhar, P.; Anselmo, A. C.; Gupta, V.; Pant, K.; Prabhakarpandian, B.; Ruoslahti, E.; Mitragotri, S., Using shape effects to target antibody-coated nanoparticles to lung and brain endothelium. *Proceedings of the National Academy of Sciences of the United States of America* **2013,** *110* (26), 10753-8.

3. Champion, J. A.; Katare, Y. K.; Mitragotri, S., Particle shape: a new design parameter for micro- and nanoscale drug delivery carriers. *Journal of controlled release : official journal of the Controlled Release Society* **2007,** *121* (1-2), 3-9.

4. (a) Irvine, D. J., Drug delivery: One nanoparticle, one kill. *Nature materials* **2011,** *10* (5), 342-3; (b) Rai, P.; Padala, C.; Poon, V.; Saraph, A.; Basha, S.; Kate, S.; Tao, K.; Mogridge, J.; Kane, R. S., Statistical pattern matching facilitates the design of polyvalent inhibitors of anthrax and cholera toxins. *Nature biotechnology* **2006,** *24* (5), 582-6.
